# Outlook of women in science: an interview with our authors

**DOI:** 10.1002/1878-0261.13189

**Published:** 2022-03-01

**Authors:** Wiktoria Blaszczak, Asma Ahmed, Katharina Leithner, Antonia Schubert, Michelle Leech, Claudine Bonder, Ioannis Tsagakis

**Affiliations:** ^1^ 6396 Department of Physiology, Anatomy & Genetics University of Oxford UK; ^2^ Cancer Research UK Beatson Institute Glasgow UK; ^3^ Institute of Cancer Sciences University of Glasgow UK; ^4^ 31475 Department of Internal Medicine Medical University of Graz Austria; ^5^ Division Signaling and Functional Genomics German Cancer Research Center (DKFZ) and Heidelberg University Germany; ^6^ National Center for Tumor Diseases (NCT) Medical Oncology Heidelberg Germany; ^7^ Discipline of Radiation Therapy Trinity St. James’s Cancer Institute Trinity College Dublin Ireland; ^8^ 1067 University of South Australia Adelaide Australia; ^9^ Molecular Oncology Editorial Office Cambridge UK

**Keywords:** equality in science, female researchers, gender equality, gender gap, women in science, women in STEM

## Abstract

Diversity in research teams ties alternative perspectives into research projects, and this can fast‐forward scientific progress. Concerted efforts have been aimed at encouraging and supporting women to pursue a career in science, yet a gender disparity can still be observed at senior positions, with fewer women in leadership roles. To get insight into how the current landscape for women in science is perceived by different career stages, we interviewed female authors of *Molecular Oncology* from diverse career stages and disciplines about their inspiration, challenges they have faced as scientists as well as their thoughts on how gender diversity can be further enhanced.

## Graphical abstract

Despite funding schemes and support networks addressing gender imbalance, female researchers still occupy considerably fewer senior positions than male scientists. We interviewed female authors of *Molecular Oncology* about their motivation, challenges faced as scientists, and their perspectives on reinforcing gender diversity. Responses suggested joint efforts from researchers and academic bodies are needed to even the scales.

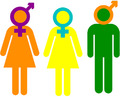

## 1 Introduction

Problem solving is a major part of conducting scientific research. Diverse gender‐balanced teams have been more effective in problem‐solving than teams composed exclusively of men or women [1]. In the last two decades, we have witnessed increasing efforts to tackle inequality across men and women in science. A clear discrepancy has been reported between male and female academics, in terms of funding outcomes [2, 3, 4], publication output and citation patterns [5, 6, 7], gaps in salaries [8, 9, 10] and uneven representation at higher ranks [11, 12]. Hence, there is a clear need for initiatives aimed at improving the ranking of women in science. As a result, women‐specific funding schemes have been launched, unique mentoring and leadership training oppor1tunities for women have been established at universities, and peer‐support networks for female scientists as well as mothers in science have emerged (Table 1). Additionally, gender quotas in research funding programmes have been introduced and encouraged by policymakers [13, 14]. Currently, there is no shortage of inspiration for women choosing to study science [15]. Nonetheless, inspiration alone is not enough to keep women in science.

**Table 1:** Examples of funding, mentoring and training opportunities available to support women and parent scientists in Biology.

Career stage	Type of support	Initiative	Impact
Towards professorship	Professorship	Ministry of Science, Research and the Arts MWK – Margarete von Wrangell habilitation programme for women	Supporting outstanding female scientists on their way to a professorship through employment relationships, with the opportunity to teach and the right to lead doctoral students
Lecturer, senior lecturer or reader level	Fellowship	RAEng/Leverhulme Trust Research Fellowships	Covers the salary costs of a replacement academic who will take over the awardee’s teaching and administration duties for up to 1 year, allowing the awardee academic to concentrate on full‐time research
PhD and postdoctoral research	Fellowship	L'Oréal‐UNESCO & Christiane Nüsslein‐Volhard‐Stiftung	Grants to support excellent female doctoral and postdoctoral researchers with children from the fields of experimental natural sciences and medicine
Postdoctoral research	Fellowship	L'Oréal‐UNESCO Women in Science postdoctoral fellowships	Recognises the achievements and contributions of exceptional female scientists across the globe
	Fellowship	Royal Society Dorothy Hodgkin Fellowships	Fellowship that offers flexible working pattern due to personal circumstances such as parenting, caring responsibilities or health issues
	Fellowship	NIHR, BHF, Wellcome Trust Career Re‐entry Fellowships	Opportunity to re‐establish postdoctoral scientific careers after a continuous break from research of at least 2 years
	Professorship	Brigitte Schlieben Lange Programme of the Federal State of Baden‐Württemberg for Female Junior Scientists with Children	Addressed to women with children who want to combine their scientific qualifications with family and professional tasks, and who would like to start or continue their scientific work
	Personal growth training	Leibniz Mentoring	Structured and supervised mentoring partnership, offering competence seminars as well as professional assistance during the process which guarantees the transfer of acquired knowledge and skills into the daily routine
Master's and early academic fellowships	Scholarship	British Council scholarships for women in STEM (Americas, South Asia, South East Asia, Egypt, Turkey and Ukraine)	Financial support aimed at women to pursue careers in STEM
College undergraduate	Award	AWIS Opportunity Scholarships for Career Re‐entry	Women‐focused personal development and leadership programmes
All career stages	Personal growth training	Athena SWAN and Advance HE	Women‐focused personal development and leadership programmes
	Fellowship	MRC Daphne Jackson Fellowships aimed at researchers returning from a career break	Funding for short‐term technical assistance or buy‐out of teaching duties, purchase of consumables/facilities access, help with costs or logistics of conference attendance or training courses (new skills or refresher training)
High school	More information soon	L'Oréal For Girls in Science programme	Offers the opportunity to join a support programme throughout the school year, which includes participation in a scientific challenge, a scientific trip, inspiring meetings, cultural outings and visits to companies

The current academic pipeline is leaky, with several researchers every year deciding to pursue research outside of academia or considering a different career altogether. Whilst there is an interest in young women to pursue a career in a science, technology, engineering and mathematics (STEM)‐related subject, gender imbalance is present even at the PhD level, with different proportions of women across STEM fields in the USA [14, 16, 17]. Moreover, the proportion of women in senior positions at the university level such as full or associate professors, remains low, indicatively ranging from 20% to 29% in the UK [11], Germany [12] and Nordic countries [18]. In Germany, only 10% of professors at university hospitals or clinic directors are women, indicating a poor representation at leadership roles (Deutsche Ärztinnenbund – German Medical Women’s Association) [19]. Besides the effect this has on the current generation of female scientists, a lack of representation of women in STEM fields could deprive young girls of role models in science, perhaps dissuading them from pursuing a science degree [20]. Therefore, when it comes to achieving equality in science, there is still room for improvement given the persisting gender imbalance at more senior positions.

For this year’s International Day of Women and Girls in Science (February 11), we asked female authors of *Molecular Oncology* from diverse career stages and disciplines to share their thoughts on what universities and funding bodies can do to support aspiring and existing female scientists (Box 1), as well as to highlight their own advice and sources of inspiration.

Box 1 **Summary of measures suggested to address inequality in research**

- Accessibility to mentoring, career guidance and workshops/seminars aimed at normalizing failure and ways to learn and grow from it.
- Systematic practices to improve equality in grant funding across all positions.
- Countermeasures to short‐term contracts which can jeopardise career stability, disproportionately affecting women.
- Mutual parental leave: Both parents should be eligible to apply for leave due to upcoming pregnancies, and male scientists should be encouraged to take it.
- Initiatives to promote active participation of men in childcare (which acts in favour of supporting women in science).
- Flexibility on funding schemes for parents in academia to allow returning to work on a flexible/part‐time basis.
- Representation of female academic researchers and mothers in science in leading academic positions, empowering them as role models in the eyes of aspiring students and emphasizing this career trajectory as a viable prospect.
- Enforcement of a family‐friendly working environment with flexible day‐to‐day working hours.
- Provision of reasonable childcare services for scientists that are parents.
- Option for group leaders to set up shared leadership teams.
- Availability of administrative and/or technical support to reduce workload for scientists with caring responsibilities or scientists on parental leave.
- Strong recommendations for academic review boards not to regard gaps in the CV due to parenting a drawback.
- Encouragement of meetings to be held within normal working hours, in consideration of scientists with childcare responsibilities.

**Question**: How could young women be inspired to enter your field?

**Claudine Bonder**: I believe that young women are already inspired to enter the field of medical research; the problem lies in retention. The overwhelming lack of job security and senior positions is what fails to keep them in science. It’s important for women to hold leadership positions so that the most diverse expertise can contribute to problem solving. If young women scientists don’t see women in senior leadership roles, they won’t know that there is any job stability or opportunity to advance.

**Wiktoria Blaszczak**: I agree that young women are inspired and they do enter the field. However, improving working conditions and gender equality would encourage more of them to stay in academia and continue their research.

**Katharina Leithner**: At present, we are 80% females in our group; I do see a great interest of young women in medical sciences and biology. To keep talented young women in science, it takes a supportive atmosphere and examples/role models of women successfully finding their way in science.

**Antonia Schubert**: In my opinion, seeing more women, especially in leadership positions and having relatable role models and mentors will not only inspire more and more young women to pursue a career in STEM but also it will happen organically.

**Michelle Leech**: Highlighting diverse and relatable role models in the field of radiation oncology would inspire young women to join the field, as would mentorship. However, probably the most inspirational aspect of our field is the ability to work with and treat cancer patients of every background and to support them on their cancer journey. There really is nothing more inspirational or rewarding than that.

**Asma Ahmed**: I think by promoting and empowering more women in academia, showing their stories, and sharing their experiences on how they overcame the challenges, young women need to feel supported to be inspired to join academia. Networking with senior female academics is key (Box 2).

Box 2 **Biographical sketches of contributing authors**

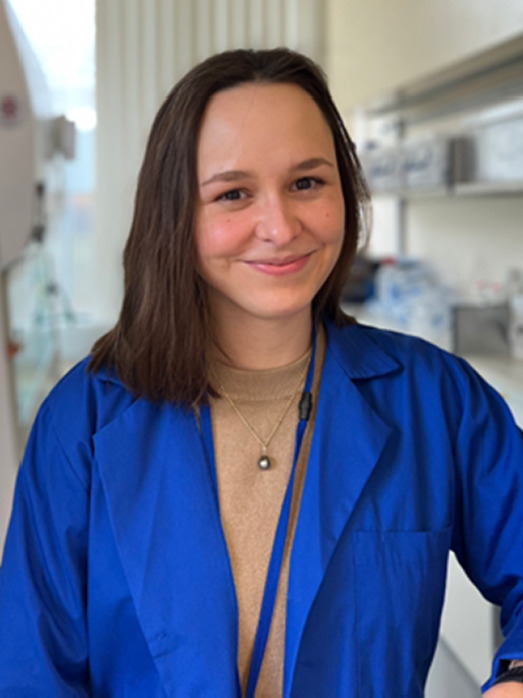

Wiktoria Blaszczak is a PhD student in the Department of Physiology, Anatomy and Genetics at University of Oxford. Her research focusses on the interplay between pH regulation and metabolism in Pancreatic Ductal Adenocarcinoma with a particular interest in cell to cell heterogeneity and its impact on disease progression. Her project is funded by the Marie Sklodowska Curie Innovative Training Network and is part of the pH and Ion Transport in Pancreatic Cancer consortium (pHioniC). This consortium unites 12 European institutions with the common goal to describe, characterize and utilize the link between pH and carcinogenesis in the clinical approach to treat pancreatic cancer.

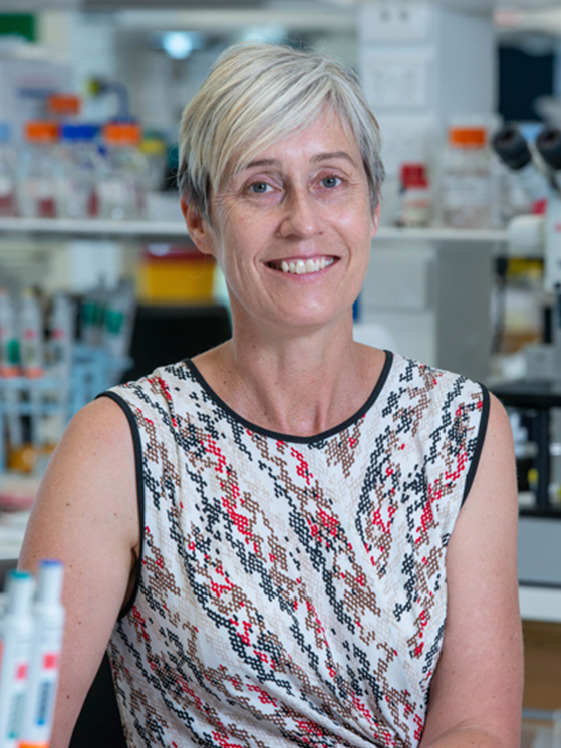

Professor Claudine Bonder is Head of the Vascular Biology and Cell Trafficking Laboratory at the Centre for Cancer Biology in Adelaide, South Australia. Her research interests focus on understanding the blood vasculature in diseases such as cancer, heart disease and diabetes. Claudine currently leads a team of enthusiastic and dedicated staff and students across a broad programme with established academic and commercial collaborators in Australia, the United States and Europe. Under her leadership, ~11 M in grant funding has come to South Australia, six patents have been filed, and new partnerships with multinational companies have been formed. Claudine is a Senior Scientific Advisor to Carina Biotech Pty (a company that develops novel CAR‐T cells to combat solid tumours) and TekCyte Pty (a company that makes medical devices BIOINVISIBLE).

In recognition of her achievements, Claudine has been awarded an Early Career Research Award from the Australian Academy of Science, a Young Tall Poppy award, an ASMR Leading Light finalist and she was the Women in Innovation SA – Emerging Innovator for 2016.

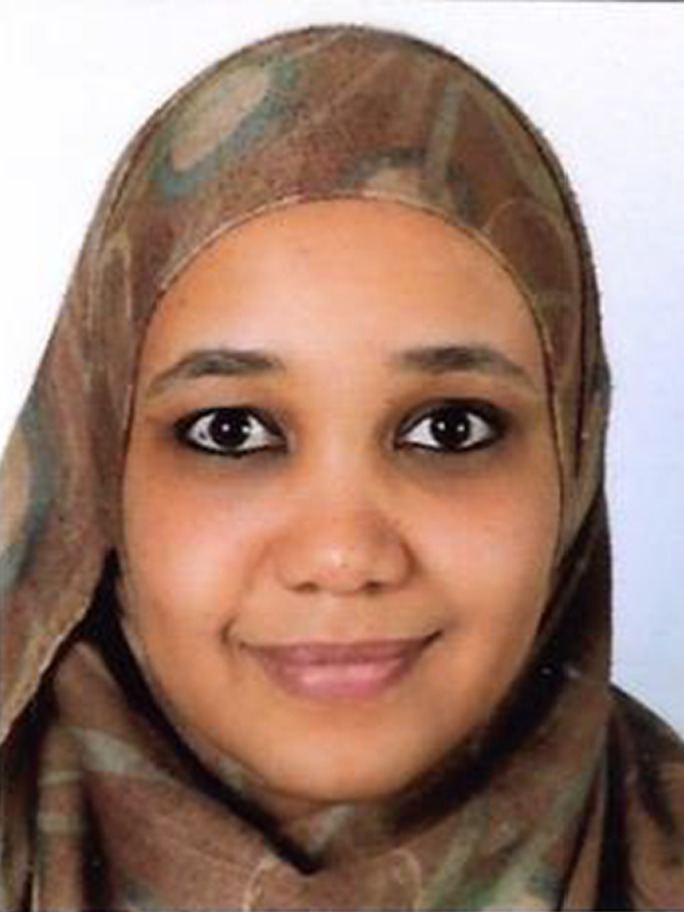

Asma Ahmed Hassan Elshiekh is a postdoctoral researcher at the Research Institute of Cancer Sciences and CRUK Beatson Institute. She became passionate about research and teaching during her initial training as a pharmacist. Therefore, she joined the Department of Pharmacology at the University of Khartoum, Sudan, as a Teaching Assistant. During her Master’s degree at the Department of Pharmacology, University of Khartoum, she developed an interest in understanding the mechanism of action of drugs, and then went on to carry out PhD studies in Germany, expanding her knowledge about tumour immunology and cancer biology at the group of Professor Thomas Brunner, Chair of Biochemical Pharmacology at the Department of Biology, University of Konstanz, Germany.

Her research on novel therapeutic targets in colorectal cancer and cancer immune evasion mechanisms led her to study cell death and tumour immunology at the group of Professor Stephen Tait. Her postdoctoral research project addresses targeting immunogenic cell death for the treatment of prostate cancer aiming to provide a novel adjuvant therapy that will hopefully help to treat advanced prostate cancer, an incurable disease with poor prognosis.

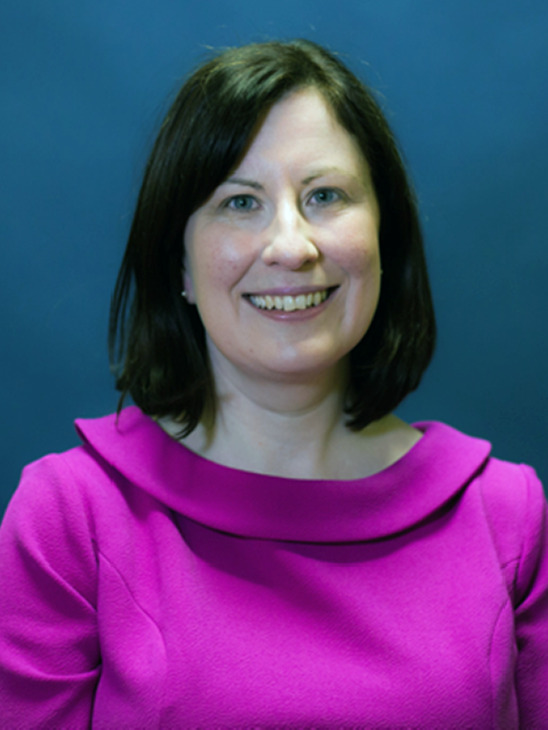

Dr. Michelle Leech is an Associate Professor at the Discipline of Radiation Therapy, School of Medicine, Trinity College Dublin, Ireland. She is Director of Radiation Medicine Education in the Discipline and is Associate Director of Health Policy and Engagement in the School of Medicine. She is past Chair of the Radiation Therapist Committee within the European Society for Radiotherapy and Oncology (ESTRO). Within ESTRO, she is chair of blended learning at the ESTRO School and is a member of both the Scientific and Educational Councils.

Dr. Leech is Editor‐in‐Chief of the ESTRO Journal *Technical Innovations and Patient Support in Radiation Oncology* and is a member of the editorial board of *`Radiotherapy and Oncology’*. She also contributes to activities of the International Atomic Energy Agency (IAEA), with whom Trinity College Dublin has a practical arrangement, particularly in the education of RTTs. Dr. Leech completed her PhD in radiomics in prostate cancer. Her current projects are focussed on radiomics, management of osteoradionecrosis in head and neck cancer, and educating patients and carers about radiation therapy as a cancer treatment.

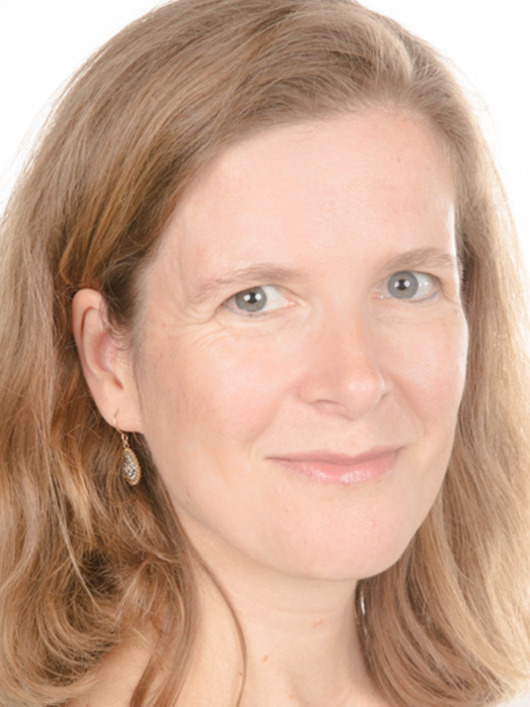

Katharina Leithner, MD, PhD, is a junior faculty member at the Medical University of Graz, Graz, Austria. Her research group studies gluconeogenesis in lung cancer cells and the mechanisms of adaptation of cancer and stroma cells to low nutrient conditions. After completing her MD studies at the Medical University of Vienna, Austria, she joined the Department of Pulmonology at her current institution, where she worked clinically for 1.5 years and then obtained a PhD in Molecular Medicine. Independent third‐party funding allowed her to start her own research group 6 years ago.

Until now, two PhD students and several master students completed their studies under her supervision as the main supervisor. Her work has been cited more than 860 times, and she is a reviewer for numerous high‐impact journals.

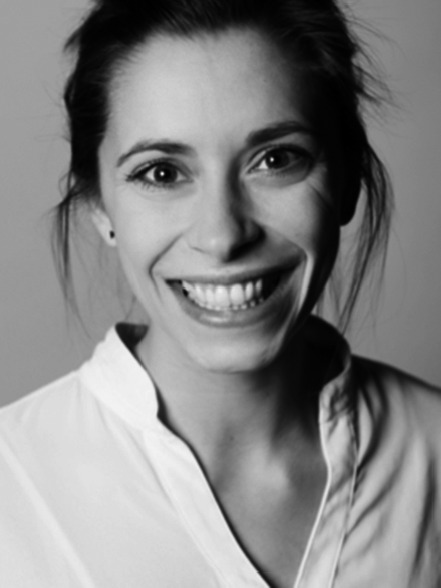

Antonia Schubert, MD, currently works as a physician scientist in the Department of Medical Oncology at the National Center for Tumor Diseases (NCT) and at the German Research Center (DKFZ) in Heidelberg, Germany. She obtained her medical degree at the University of Göttingen in 2015 and her Doctorate of Medicine in 2016, investigating the roles of Wnt signalling during breast cancer progression and metastasis.

After completing her basic medical training at the University of Göttingen in the department of Hematology and Medical Oncology, she joined the lab of Prof. Michael Boutros at the DKFZ and Heidelberg University as a postdoctoral researcher in 2018, in part supported by the Collaborative Research Center 1324 on Wnt signalling.

She investigates the roles of extracellular vesicles (EVs) in cancer with a focus on EV‐mediated Wnt signalling. The ultimate aim of Antonia Schubert’s research is to broaden our mechanistic understanding of EV‐mediated communication and translate the research findings into clinical applications. Since 2020 she continues her clinical residency at the NCT, Heidelberg in parallel to her scientific work in the lab of Michael Boutros. She is part of the DKFZ Clinician Scientist Fellowship Program supported by the Dieter Morszeck Foundation.

**Q**: What inspires you?

**AA**: I am inspired by our contribution to the generation and sharing of knowledge. Also hearing success stories and learning from other people’s experiences.

**WB**: I feel inspired by the translational value of my work. The potential application is what makes it meaningful to me.

**AS**: I work as a Clinician Scientist in basic research and medical oncology in Heidelberg, Germany. During my medical training, I was inspired to enter the scientific world and pursue a dual‐career by female role models. Additionally, I found in science what I had been missing in the hectic, daily clinical routine: the opportunity to explore and understand physiological and pathophysiological processes and a continuing intellectual challenge. Whilst it is an extraordinary privilege and responsibility to work as a clinician, I enjoy the possibility of combining clinical work with curiosity‐driven science. Using all this to potentially improve patient outcomes at some point is a great inspiration and motivation for me.

**KL**: The discoveries of unexpected, yet unknown features of biological systems or diseases and the people behind them. On the other hand, the feeling of cooperative work and the sharing of data and ideas is encouraging and inspiring to me. I am happy that I can experience this frequently in my research field and at my institution.

**ML**: Professionally, I am inspired by working with colleagues across disciplines and valuing the contribution that every discipline and every person makes to a collaborative project. One of the most inspiring aspects of such work for me is being challenged to think in a completely different way about a methodology or a result and seeing it from a totally different perspective.

**CB**: I am constantly amazed and inspired about the complexity and intricacy of cell biology. Being able to investigate how cells contribute to cancer fascinates me, and what drives me is knowing that I have the skill‐set to drill down and unpack this process so that better prognostics or treatment options can be developed. I absolutely love my job; I get to work with the most amazingly talented team of people who work tirelessly during the week and then dedicate extra time at night, over the weekend and their holidays to improve the health (and save the lives) of complete strangers. A career in medical research is different every day; it’s filled with problems to solve, some of which are small and quick to complete, others are huge and require dedication, lots of time and an internationally diverse team of experts to complete.

**Q**: What are the main challenges about being a female scientist in your field?

**WB**: At this stage of my career, I feel most challenges are not gender‐related. We all sometimes struggle with experiments, worry about funding or try to tackle not very promising manuscript revisions. Depending on the community in a workplace, however, it can be difficult to feel properly heard as a woman, especially in early stages of the career. Men tend to be perceived as more confident, competent and trustworthy. Therefore, I think the challenge is not only to shift this attitude but also to ensure not to believe in this opinion oneself.

**AS**: According to the Deutsche Ärztinnenbund (German Medical Women’s Association), more than 60% of medical students in Germany are female [19]. Still, only 10% in top clinical positions, such as professors at university hospitals and directors of a clinic or institute, are women. This ratio is similar in other natural sciences, such as biology. Only a few female medical doctors choose and stick with the dual‐career and leadership positions. One reason is competing interests from clinical and research institutions and family responsibilities, especially at the transition from residency to independent faculty positions which often coincides with becoming a parent. Parental leave and a potentially divided focus during a time period that is crucial for a scientist’s career development are major challenges – but not necessarily for females only. Still, the way motherhood and maternity leave are perceived by supervisors and colleagues of both genders poses barriers to gender equality. Nevertheless, the increasing awareness that a diverse workforce including women in leadership positions grants the crucial skills and experience required to drive innovative research and improve patient care is changing the current landscape. Despite these challenges, working as a Clinician Scientist is incredibly rewarding. Achieving the compatibility and positive leverage of the two careers whilst having a fulfilling private life is a prerequisite for women to be able to choose a dual‐career track.

**KL**: Luckily, I experienced a lot of support by my supervisors and bosses. The challenges emerged from the fact that combining clinical work and research or spending a long time abroad is difficult with small children. Faced with these challenges, I made the decision to completely switch to research and quit the clinic, in order to actively pursue international collaborations with shorter lab visits, respectively. I think a lot of progress has been made in research to support females, for example, through special funding schemes for women. However, institutions and grant agencies must continue to critically assess any systematic disadvantage for women in an ever‐changing society. Nevertheless, women and men face similar difficulties when applying for grants, establishing or maintaining a research group, and the path towards a permanent position in academic research is often hard. But it is worth trying.

**ML**: The main challenge in radiation oncology is the lack of awareness of it as a specialty, and this applies to all of us who work in this field. Surgical and medical oncology are well known and understood, but many misconceptions still surround radiation oncology, which can make funding of radiation oncology projects difficult, even if the field has a good gender balance.

**AA**: The main challenge in my field is balancing caring responsibilities and family commitments with a demanding workload.

**CB**: Job security and being recognized as a competent expert by our peers. Despite recent times wherein we have witnessed first‐hand the need for urgent and scientific rigour to manage a global pandemic, Australian scientists continue to fight for job security and basic funding. The Australian Society for Medical Research recently completed a survey showing that 1 in 4 Australian researchers do not have job security for the following year, and for women (who are a minority here), the challenge is even greater.

**Q**: In your opinion will the pandemic impact gender equality in science?

**WB**: I hope the pandemic will have a positive impact on gender equality in the end. Multiple lockdowns and an obligation to work from home proved that in many cases, work can be done as efficiently from home as it would have been done from the office. Hopefully, the newly adopted flexibility in terms of working hours and/or working on site will improve the work‐life balance of people with childcare responsibilities, which often are women.

**KL**: I believe that women will overcome any problems arising from hampered childcare, closed schools, etc. And I am confident that the pandemic will lead to a rise in awareness that the society needs scientists.

**AS**: Women (as other minorities in STEM) suffer more drastically under the ongoing COVID‐19 pandemic, which enhances pre‐existing inequalities. Systems put into place to guarantee the proper work‐life balance, such as childcare services and flexible work arrangements – both essential for productivity and research success – fell apart within days. In most cases, women took over the unpaid care work and associated mental load – not only during lockdowns. For some, this had a significant negative impact on their mental health. Procedures and practices promoting inequitable gender roles and norms reinforce unbalanced power dynamics and hinder lasting change through female expertise and experience.

**AA**: The main impact of the pandemic is related to the increased caring responsibilities of female scientists. From my own experience as a mother of two school children, I found it very challenging and stressful to help my children with their online learning and at the same time do my own work.

**CB**: There is no doubt that this pandemic will severely impact gender equality in science. For example, because women continue to hold the majority of carer responsibilities for their young and the elderly, the additional time required to supervise remote learning, organize vaccinations and attend to heightened levels of anxiety within the home, will undoubtedly cause their productivity to suffer. Unless we can change the current narrative, the current stereotypes and bias in science will mean that a consequence of this pandemic will be that women will be further disadvantaged whilst men continue to rise to the top.

**ML**: The pandemic has had very difficult consequences for individuals in every field. For example, those with caring responsibilities, be it mothers and fathers, other relatives or children, have often had disproportionate workloads to balance during the waves of the pandemic. In academia, this has impacted conclusion of projects and outputs. Funders who have not permitted extensions of grants under these circumstances have put additional stressors on scientists as well as institutions who have not adjusted the metrics of expected outputs for such individuals accordingly. Everyone has their own story and own situation to contend with from the pandemic, so showing a little compassion and pragmatism during this time will ensure that all excellent scientists will wish to remain in their fields when the pandemic has passed.

**Q**: What measures should be put in place to support women in academia?

**ML**:

In my opinion, when entering academia, one of the most important measures for any individual, however they identify, is to normalize ‘failure’ and to take every perceived ‘failure’ as an opportunity for growth and development.

We should try to see something positive in even the most challenging experiences; I remember having my first manuscript rejected and feeling completely deflated, not realising that this would be a regular occurrence throughout my career! *‘Learning from every event’* seminars would be a positive step forward on induction into an academic career to support this.

**WB**: Many women enter academia; however, data show that significantly fewer women than men reach higher positions, as Principal Investigators or Professors. My observations indicate that despite promoting gender equality, women are still mainly responsible for childcare and often feel that they are facing a choice between having a family and a professional career. I think it would be helpful to promote equality in childcare responsibilities, perhaps by introducing mutual parental leave that can be taken by either parent. In addition, universities and funding bodies could adopt programmes that would allow new mothers to come back to work on a more flexible/part‐time basis, so they wouldn’t feel deprived of experiencing their child development.

**AS**: Several steps have been taken in the last years to support women in academia, especially in western countries. Girls and young women are increasingly interested in STEM subjects. Funding opportunities for financial support and mentoring programmes tailored to the needs of women have been established at various biomedical institutions in Germany. Nevertheless, female role models in leading academic positions are still scarce, with scientists that are mothers remaining underrepresented. This absence of representation will lead to the conclusion that ‘one just simply cannot have it all’. Competing family responsibilities, lack of flexible childcare services and short‐term employment contracts force highly educated and talented female scientists off the research ladder to find more flexible and stable job alternatives. For me, supporting women in academia is more than just compensation at the entry level to get the girls interested in STEM. It's about fixing the leaking pipe and keeping them there, for example, by providing a family‐friendly working environment through flexible working hours, reasonable childcare services, options for shared leadership, administrative or technical support for employees taking over care work.

**KL**: There should be rules or recommendations for academic review boards that any gap in the CV that has been caused by parenting is not regarded as a drawback, for example, when applying for a PhD position, junior faculty member position, etc. This applies to both, mothers and fathers.

The active participation of men in childcare is the most important factor for the support of women in science and elsewhere, and if time for parenting is regarded as a necessity, which does not hamper scientific abilities, it will improve the life of both, male and female scientists.

Moreover, institutions should support and provide childcare and actively encourage PIs or department chairs to avoid meetings outside normal working hours.

**AA**: I believe there is still much to be done to support women in academia, and I am glad that this issue is now in the spotlight and there are some efforts to improve it. One important measure would be to support flexible working practices and provide mentoring and career guidance for women to reach their full potential. Moreover, supporting women scientists, especially those with families and children, with a research technician to share the workload, for instance, could help them find the right life/work balance. Another example would be providing childcare at the place of work. I have attended an online career development workshop during lockdown, and it was shocking to hear that most female researchers are finding it difficult to advance in academia due to caring responsibilities and the lack of support from their employers and, of course, the demanding nature of our job.

**CB**:

Thanks to those who paved the way before us, there has never been a better time to be a woman in science; but we still need to challenge the current stereotypes and fight the bias that prevents us from reaching our full potential.

Scientists in general (but women in particular) need much better job security, more career stability and gender equality in senior positions. I believe that society underestimates the skills that are required to be a scientist; it takes years of specialist training to work independently in a laboratory and more dedicated mentoring to be able to lead a research team. Without job security, all of this training is lost to the sector, life‐saving scientific discoveries simply vanish, and there is no next generation of scientists. We need to fight to keep them. Women in science would undoubtedly benefit from equality in grant funding, fellowships and senior leadership positions; the rest will follow. Two support measures that Australia could implement would be (i) shared/equal paternity and maternity leave to allow women to return to work earlier and (ii) equality on grant funding and fellowships.

**Q**: What advice would you give your younger self?

**CB**: I will be forever thankful to everyone who has helped shape and guide my scientific career as I absolutely love what I do. But if I had a chance to speak to my younger self, I would reassure her that you don’t have to be one of the boys to sit at the table, don’t be afraid to speak up against gender bias, seek out a female mentor who could provide constructive advice on the challenging road ahead, and remember to elevate others at every chance.

**AA**: Family time is precious, so try to spend more time with your family. Work hard but don’t forget to enjoy life as well. Find the right work/life balance.

**AS**: Be patient – good things take time. As physicians working in the clinic, we are used to immediate feedback. Working in basic science, projects can stretch over several years. When periods with protected research time and medical training are protracting each other, stamina is indispensable.

**KL**: To keep critically believing in yourself and your abilities and to stay positive. Importantly, to see that progress in science needs the expertise and commitment of many people around you.

**WB**: I would tell myself not to feel discouraged and defeated by my failures as they are a part of life, especially in academia. The idea should not be to avoid failure, but to derive information from it.

**ML**: Take every opportunity that comes your way and be prepared. You make your own luck and when an opportunity arises, as it inevitably will, be ready and deliver your work to an exceptional standard. Be known for excellent work, and others will want to work with you, this increases your network and your reach.

## Conflict of interest

The authors declare no conflict of interest.
